# PotatoRTD and TomatoRTD: Comprehensive Reference Transcript Datasets for Accurate Transcriptome Analysis and Isoform Discovery

**DOI:** 10.1038/s41597-026-07792-1

**Published:** 2026-08-24

**Authors:** Wenbin Guo, Linda Milne, Sara Lopez-Gomollon, Amanpreet Kaur, Ingo Hein, David Baulcombe, Runxuan Zhang

**Affiliations:** 1 Department of Information and Computational Sciences, The James Hutton Institute, Invergowrie, Dundee, Scotland UK; 2 Department of Plant Sciences, University of Cambridge, Cambridge, CB2 3EA UK; 3 Department of Cell and Molecular Sciences, The James Hutton Institute, Invergowrie, Dundee, Scotland UK; 4 International Potato Center (CIP), Lima, Peru; 5 School of Life Sciences, University of Dundee, DD1 4HN Dundee, Scotland UK; 6 Present Address: Higentec Breeding Innovation (ZheJiang) Co., Ltd., Lishui, China; 7 Present Address: School of Natural Sciences, University of Kent, Canterbury, United Kingdom

## Abstract

Transcriptome annotations provide essential information on transcript locations, sequences and structures, including transcription start, end sites and splice junctions. They underpin key biological analyses such as gene and transcript quantification, and the study of transcriptional and post-transcriptional regulation, including alternative transcription initiation, polyadenylation and splicing. Accurate characterisation of transcript isoforms is critical for understanding how gene expression relates to functional protein products. However, for many species—including Solanaceae crops such as potato and tomato—current annotations suffer from limited isoform coverage, with tens or hundreds of thousands of splice junctions and transcript isoforms missing. This undermines the completeness and accuracy of transcript-level analyses. Here, by generating Iso-seq and RNA-seq on a range of tissues and samples, we have produced transcriptome annotations for both potato and tomato with improved coverage, diversity, accurate splice junctions, and transcript start and end sites. We have also made these high-quality resources accessible through genome browsers. These enhanced annotations will enable more accurate transcriptome analyses, supporting higher-resolution and novel biological discoveries.

## Background & Summary

The transcriptome annotations of a species provide key information on the location, sequence and transcript structures (start, end and splice junctions). They often consist of information on definitions of exons and introns, untranslated regions (UTRs), alternative isoforms and the coding sequences (CDS) of individual genes. Together with the reference genome sequence, they are the most important resources for genetic analysis and molecular biological research. Accurate annotations are required for genome editing and biotechnology. Applications such as CRISPR or PCR rely on knowing exon-intron boundaries, coding regions, UTRs and isoform structures. Poor annotation can lead to incorrect guide design and possible off-target effects and misinterpretation of results. They also determine how the gene expression is measured and interpreted as most RNA-seq and transcriptomics tools, such as Salmon^1^, Kallisto^2^, depend on the annotated transcript models. Incomplete or inaccurate annotations can lead to erroneous read mapping, thereby biasing transcript abundance estimates and compromising the reliability of differential expression analyses^3–5^. This limitation is particularly consequential for studies of alternative splicing (AS) and transcript isoform function. Incomplete transcript annotations obscure true biological complexity, leading to undetected splicing events and the mischaracterisation—or complete omission—of isoform switching, where two major transcript isoforms change their relative abundance in response to a perturbation.

One key issue with the transcriptome annotations for most of the plant species is the low coverage of transcript diversity. The gene/transcriptome annotations are usually produced by computational pipelines that are protein-coding-sequence-focused and provide only one or two transcript isoforms per gene. Recent studies based on long read sequencing show that the transcript isoform per gene is much richer than current annotations or assembled by short read sequencing technologies indicate^6–8^. It is known that individual transcript expression can be condition- or tissue-specific^8^. To capture the spectrum of transcript isoforms, samples covering various tissues and conditions are required. An approach to improve the coverage of transcript isoforms in transcriptome annotations is to assemble transcripts using computational tools such as Cufflinks^9^, Stringtie^10^ and Scallop^11^ from various short and long read sequencing projects that covers a diverse number of organs, tissue and conditions. According to various benchmark studies, up to 50% of the transcripts produced directly by these tools, particularly transcript assemblies based on short reads, could be mis-assembled^12,13^, Subsequently, these are manifested as transcripts containing false splice junctions, incomplete transcript fragments, or transcripts with over-extended or missing 3′ and 5′ regions. These mis-annotations have a significant impact on the accuracy of transcript and gene quantifications using RNA-seq data when using tools such as Salmon or Kallisto.

Over the past ten years, we have developed several computational methods to address the above-mentioned issues for short-read and long-read sequencing data^14–16^. These methods have been applied to several plant species, including ***Arabidopsis***^15,16^, barley^17,18^ and lettuce^19^. Our studies have shown that the new transcriptome constructed using these methods is more accurate and more comprehensive. This allows the quantification of transcript isoforms to be determined with greater improved accuracy and the gene expression analysis to be carried out at higher resolution which better captures the complexity of biology in plants^15–19^.

To produce improved transcript annotations in potato and tomato, we have generated paired short and long read sequences for 7 different tissues/conditions with substantial sequencing depth. By applying the validated computational pipeline we developed, we generated potato and tomato transcript reference datasets (RTDs), which represent transcriptome annotations with high quality and comprehensiveness. We also constructed two genome browsers to make the new annotations accessible to biologists by allowing detailed visualization and investigation of individual transcript models.

## Methods

### Potato and tomato samples and materials

**Fig. 1:**
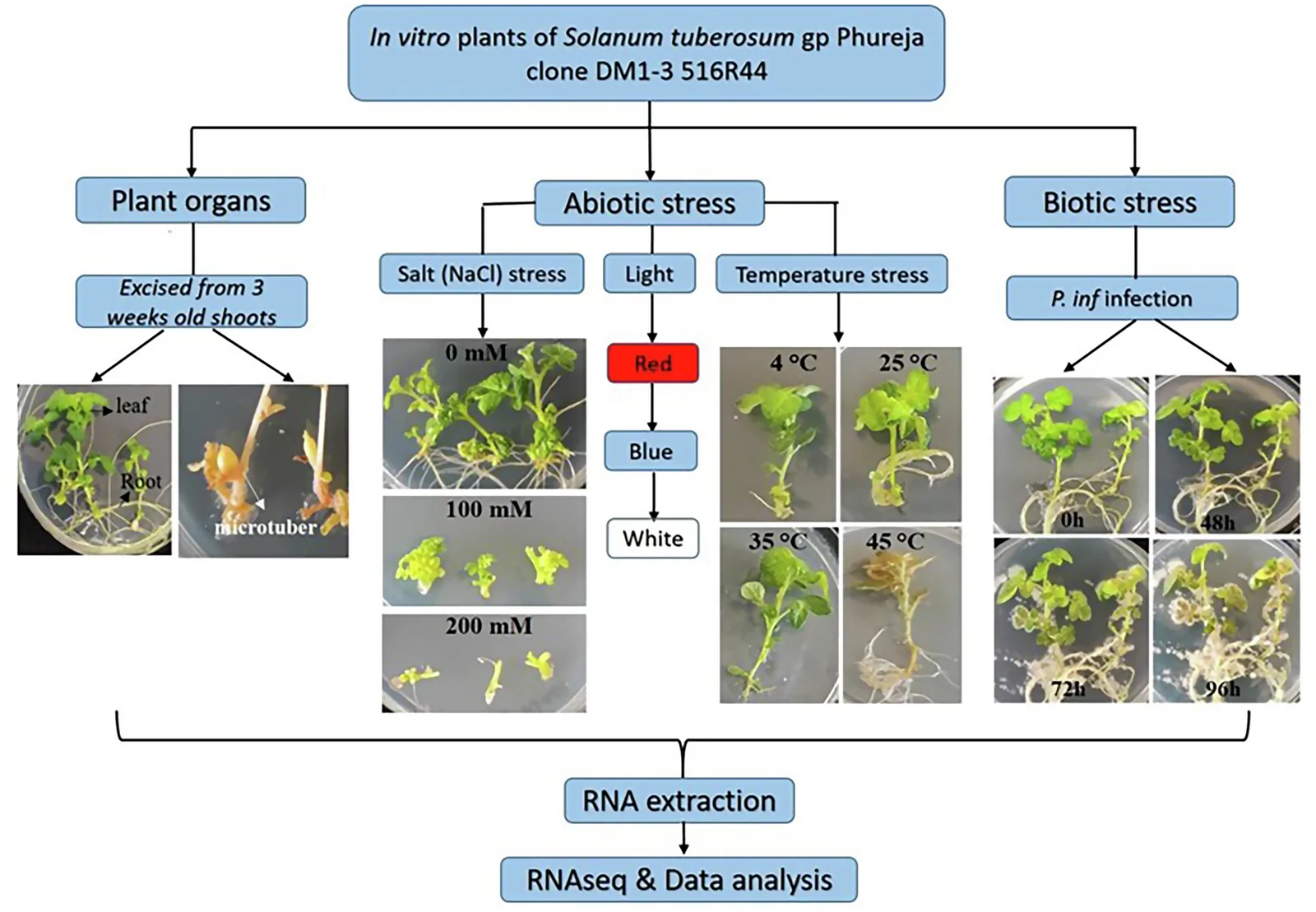
Potato Samples.

To capture organ-specific and stress-responsive (abiotic and biotic) transcripts, a total of seven RNA-seq libraries were generated using samples from *Solanum tuberosum* group Phureja DM 1-3 516 R44 (DM) (Fig. 1 and Table 1). The purpose of this study is to maximize the capture of transcript diversity, thus there is no biological replicate for any of the samples. All samples (detailed below) were immediately frozen in liquid nitrogen and stored at −80 °C until processing. Total RNA was extracted following manufacturer’s guidelines (RNeasy Plant Mini Kit, Qiagen). RNA quality was checked using Bioanalyzer 2100 (Agilent) before RNAseq library preparation.

**Table 1:** Potato samples.

Potato RNAseq library	Treatment	Description
1	Biotic stress	*Solanum tuberosum* group Phureja DM 1-3 516 R44 (DM) micro shoots were infected with *Phytophthora infestans* isolate W9928C and leaf samples were collected after 24 h and 48 h. RNA was pooled for library preparation.
2	Plant organs	Pool of RNA extracted from shoot, leaf, root and micro tubers harvested from the 3-4 week old micro shoots of DM
3	Salt stress for 15 days	Nodal segments of DM were cultured on MS20 medium containing 0, 100, 200 mM NaCl. The leaf samples and stems (for higher salt concentration, where no leaves were available) were collected after 15 days post salt stress. RNA extracted from samples were pooled for library preparation
4	Salt stress for 30 days	Nodal segments of DM were cultured on MS20 medium containing 0, 100, 200 mM NaCl. The leaf samples and stems (for higher salt concentration, where no leaves were available) were collected after 30 days post salt stress. RNA from the samples were pooled for library preparation
5	Hot and cold stress	Micro shoots of DM were subjected to a temperature shock at 4 °C, 25 °C, 35 °C and 45 °C for 24 h. RNA from these samples except 45 °C (all the micro shoots died) were pooled
6	Light treatment	Micro shoots of DM were kept under white, blue and red light for 48 h before collecting leaf samples. RNA was pooled for library preparation.
7	Light treatment and infection	Micro shoots of DM were infected with *Phytophthora infestans* isolate W9928C and kept under white, blue and red light for 48 h before collecting the leaf samples. RNA from the samples were pooled for library preparation.

For potato organ-specific library preparation, samples (leaf, root, shoot and microtubers) were collected from non-infected plants. To induce microtuberisation, nodal segments were cultured on previously formulated tuberization medium^20^ for 7 days followed by an incubation under complete darkness. Microtubers were harvested after 28 days of culture. RNA extracted from all the four organs was pooled to generate single organ-specific library. The remaining six libraries were generated from leaf tissues (and stems where indicated) collected under different stress conditions, including 1) Temperature stress: Three-week-old microshoots were incubated at 4 °C, 25 °C, and 35 °C for 24 hours. The 45 °C condition was excluded due to plant mortality. RNA from these samples was pooled to prepare one temperature-stress library; 2) Light treatment: Microshoots incubated at 25 °C were also exposed to different light conditions (blue, red and white) for 48 hours. Leaf samples were collected, and RNA was pooled to generate single light-treatment library; 3) Light treatment combined with biotic stress: Microshoots were infected with *Phytophthora infestans* isolate W9928C and incubated under white, blue and red light for 48 h. Leaf samples were collected, and RNA was pooled to generate one combined light and infection library. 4-5) Salt stress (15 and 30 days): Nodal segments of DM were cultured on Murashige and Skoog (1962) medium containing sodium chloride (NaCl; 0 mM,100 mM and 200 mM) and incubated at 25 °C with 16/8 h light/dark conditions at 80 µmol m^−2^ s^−1^. At higher salt concentrations, leaves were rudimentary, thus shoots were included for RNA extraction. Samples were collected independently after 15 days and 30 days and RNA was extracted separately to generate two pooled libraries for salt stress (15 and 30 days).; 6) Biotic stress: Microshoots were infected with *P. infestans* isolate W9928C kept and incubated at 25 °C under dark conditions. Leaf samples were collected at 24 h and 48 h post infection, and RNA from both time points was pooled to generate single biotic stress library (Fig. 1).

*Solanum lycopersicum cv*. M82 plants were grown in compost (Levington M3) and maintained (24–18 °C, 16 h/8 h day/night regime). In total, 7 samples (no biological replicates) were generated from individual tissues such as leaf, flower, fruit and root as well as partial or whole plant sampled under stress conditions, such as drought, cold and biotic stresses. The detailed description of these samples is provided in Table 2.

**Table 2:** Tomato sample details.

RNA-seq ID	PacBio ID	Tissue	Description
R1	P1	Leaf	Pool of 6 axillary young leaves (1-4 cm)
R2	P2	Flower	Pool of 20 flowers from 3 mm closed buds to open flower (covering the developmental transitions in tomato flowering)
R3	P3	Fruit	Pool of 15 fruits from 3 mm small green to 3 days after breaking (covering all developmental stages of tomato ripening)
R4	P4	Roots	Roots from 6 young plants (4week old)
R5	P5	Drought	4 week old plants were drought stressed for 1 day, 3 days and 8 days. For each time point it was collected the aerial part of 2 plants. Equivalent ground sample weight for each timepoint was combined to produce the sample used for library preparation.
R6	P6	Cold	4 week old plants were challenged with cold treatment (4 C, dark) for 2 h,4 h,24 h,48 h. The sample used for library preparation was a pool of equivalent weight of ground tissue from each timepoint.
R7	P7	Biotic	4 week old tomato plants were infected with *Pseudomonas syringae* pv. tomato DC3000 and aerial samples collected 3 dpi. The sample was a pool of two infected plants.

Samples were ground with liquid nitrogen mortar and pestle. Total RNA isolation was performed with TRIzol reagent (Invitrogen) and the Direct-zol RNA Miniprep (Zymo Research) according to the manufacturer’s instructions.

### RNA sequencing

RNA samples were pooled into seven libraries in equimass amounts, and pooled libraries were used for Iso-seq™ full-length isoform RNA sequencing and Illumina short read RNA sequencing. Full-length isoform RNA sequencing (Iso-seq™) was performed by Novogene (UK) Company Limited (Cambridge, UK) using PacBio SMRT Sequel II platform. Illumina short read transcript sequencing was performed by Novogene using Illumina NovaSeq 6000 platform.

## Bioinformatics analysis

### RTD assembly from short reads and long reads

**Fig. 2:**
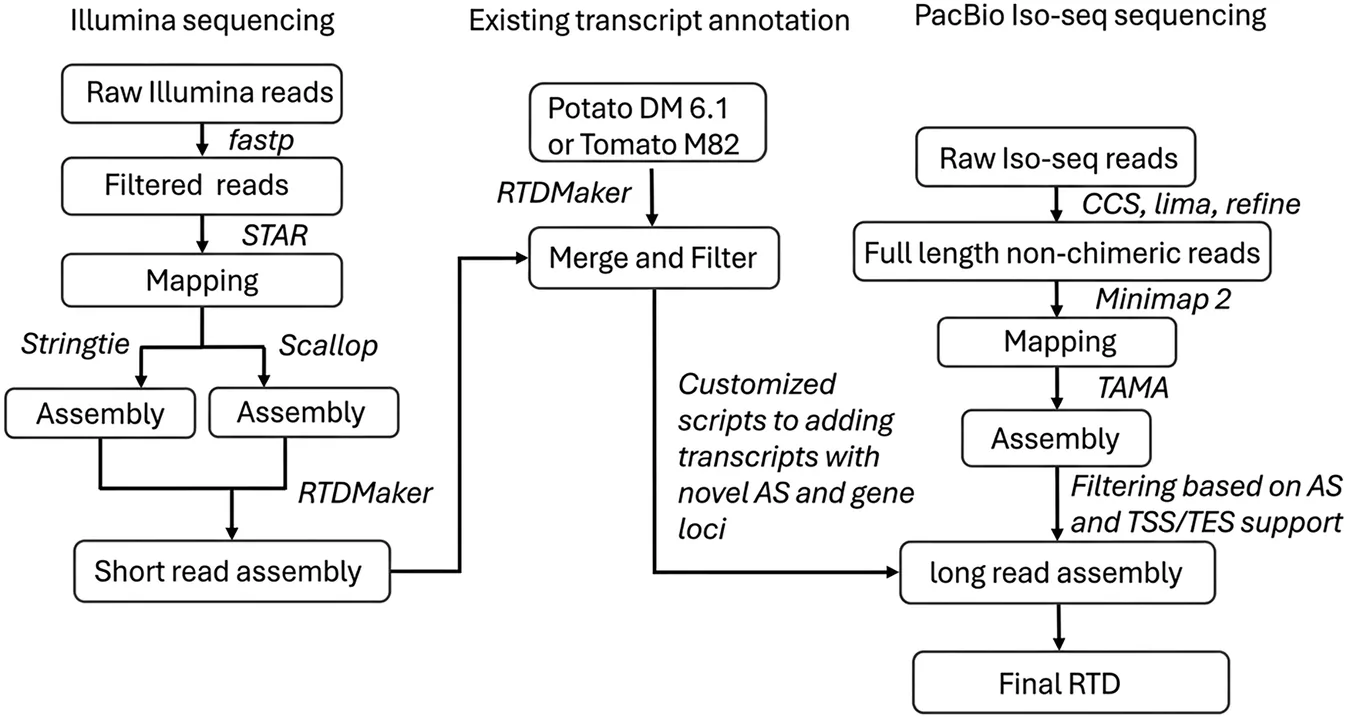
Workflow of generating the RTD for potato and tomato.

The assembly of these two high quality transcriptomes involves the transcript assembly from short reads, long reads as well as the existing transcriptome annotations. The overall analysis diagram is shown in Fig. 2.

### RTD assembly using Illumina short read sequencing data

The sequencing was carried out at ultra-deep coverage with 41.2 M pairs of reads per sample for potato with a minimum of 39.6 M pairs of reads for sample R5 (Table 3). The average mapping rate of each library to the potato genome is 93.2%, reflecting a good coverage and high quality of the transcriptomics data. For tomato, 45 M pairs of reads were generated in average per sample with an average of 97% mapping rates. The raw reads were processed with fastp v0.20.1^21^ to remove adapters. Reads with quality scores below 20 and reads shorter than 30 bp were removed. The trimmed reads were aligned separately to the corresponding reference genome for each species—Potato DM v6.1 (https://spuddb.uga.edu/dm_v6_1_download.shtml) and Tomato (*Solanum lycopersicum cv*. M82; https://solgenomics.net/) hereafter, referred to as Potato DM v6.1 and Tomato M82, respectively. Alignments were performed using STAR v2.7.8a^22^, allowing up to one mismatch, a minimum intron size of 60 and a maximum intron size of 15,000. The two-pass mode was enabled to improve the sensitivity of splice junction (SJ) detection, where junctions identified in the first pass were used as prior information in the second pass. For each sample, transcripts were assembled from the alignment files (BAM) using both StringTie v2.1.5^10^ and Scallop v0.10.5^11^. The assembled transcripts were further evaluated, filtered, and merged across samples using a short pipeline RTDmaker (https://github.com/anonconda/RTDmaker), which we developed based on our previous studies^14,15^. Filtering steps include removal of fragmentary transcripts shorter than 70% of the corresponding gene, antisense mono-exonic transcripts shorter than 50% of the sense gene, transcripts containing non-canonical splice junctions, transcripts with splice-junction read support of fewer than 10 reads in fewer than two samples, and transcripts with expression levels below 1 TPM in less than two samples. Transcriptome annotations for both species (Potato DM v6.1 and Tomato M82) were integrated into the short read–derived assemblies separately using RTDmaker (Fig. 2).

**Table 3:** Number of reads per sample for short and long read sequencing.

species	Sample Name	shorts read (Million pairs)	long read (FLNC)
potato	R1	41.5	3669933
	R2	41.5	4217662
	R3	40.5	4222505
	R4	41.6	3882824
	R5	39.6	3823905
	R6	41.2	2783946
	R7	42.3	3343106
tomato	P_1	48.3	4069087
	P_2	45.8	3504714
	P_3	46.6	4232690
	P_4	46.5	3777379
	P_5	46.7	3732980
	P_6	39.2	4089938
	P_7	42.4	3756246

### RTD assembly using PacBio Iso-seq sequencing data

The long reads were processed with IsoSeq pipeline (https://github.com/PacificBiosciences/IsoSeq). Circular consensus sequences (HiFi reads) were generated from PacBio subreads by the CCS v6.3.0 with minimum predicted accuracy 0.9 (–min-rq 0.9). Primers were removed and reads were demultiplexed using lima v2.5.0 in iso-seq mode with the peek-guess option enabled (–isoseq and–peek-guess). The reads were then processed with isoseq3, which carried out poly(A) trimming and removed concatemers. In total, 27.1 million high-quality clean reads were retained for the potato libraries (average 3.9 million per sample) and 25.9 million for the tomato libraries (average 3.7 million per sample). These cleaned reads were aligned to the corresponding reference genomes with minimap2 v2.24^23^. Transcript models were built from the aligned BAM files using TAMA^24^ collapse, and all samples were merged to make a single RTD in each species. Several filters were applied to improve accuracy and reduce redundancy. High-confidence splice junction filters removed transcripts that had mismatches and indels within ±10 nt of splice junctions, because the sequence mismatches and indels are identified as an informative indicator of false splice junctions^16,18^. To allow biological variations at 5′ and 3′ ends, transcripts with TSS/TES positions within ±50 nt were represented as a single transcript in the annotation. To remove fragmentary transcripts, we detected significant transcription start sites (TSSs)/transcript end sites (TESs) using a binomial function to estimate the probability the TSS/TES is biologically relevant^16^. We further filtered transcripts by retaining only those with significant TSSs/TESs. In addition, abundance filters excluded transcripts with fewer than two supporting reads.

### RTD merge and annotation

The long read RTDs were used as the basis to merge long- and short-read transcript assemblies as they contain the transcripts assembled with highest integrity and only transcripts containing additional coverage in terms of gene space or splice junctions are added to the final RTD. Transcripts from the short read RTD were incorporated only when they introduced novel splice junctions or novel gene loci (Fig. 2). In terms of gene name assignment, gene loci in the newly assembled RTD were compared with those in the existing transcriptome annotations (Potato DM v6.1 and Tomato M82) individually; when the overlap exceeded 30% in both datasets, the assembled transcript was assigned the gene ID from the existing annotation, otherwise it was designated as a novel gene ID. If a transcript set was located entirely within the intron of another gene, it was classified as an intronic gene model which was assigned a new gene ID. This strategy generated a high-quality RTD that combined the complementary strengths of long- and short read assemblies^16,18,19^.

### Isoform-Resolved CDS prediction and protein translation

Protein-coding sequence identification and translation were performed using TranSuite^25^ in Auto mode. Transcriptome annotations in GTF format and the corresponding transcript nucleotide FASTA sequences were provided as input. TranSuite iteratively inferred coding sequences (CDSs) and translated transcripts into protein sequences through multiple rounds of start-site correction and translation refinement to accurately identify alternative translation start sites. CDSs were first identified using a permissive ORF length threshold (≥30 amino acids) to retain potentially truncated or prematurely terminated transcripts for downstream analyses. Translated peptides shorter than 100 amino acids were subsequently excluded from the final protein set. Premature termination codons (PTCs) were defined using a cutoff of 70% of the CDS length. The iterative start-fixing and translation procedure was performed for up to five cycles to maximize CDS completeness and consistency across transcript isoforms. Transcript functional annotations were assigned using InterProScan v5.65^26^ and Panzer v1.4.1^27^.

## Data Record

All raw data files for both RNA sequencing data and PacBio Iso-seq sequencing data have been deposited at the Sequence Read Archive (SRA) under the following Bioproject number: PRJNA1412869^28^ for potato (https://www.ncbi.nlm.nih.gov/bioproject/PRJNA1412869) and PRJNA1406150^29^ for tomato (https://www.ncbi.nlm.nih.gov/bioproject/PRJNA1406150) separately.

The potato and tomato reference transcript datasets, including transcript sequences, transcript annotations in GTF format, protein translations in FASTA format, and functional annotations generated by InterProScan and PANNZER, have been deposited in Zenodo under 10.5281/zenodo.20160822 (https://zenodo.org/records/20160822)^30^.

## Technical Validation

### Checking transcript annotations in their genome coverages, AS and transcript diversity

The construction of PotatoRTD and TomatoRTD markedly expanded genome coverage, AS and transcriptome diversity compared with current transcriptome annotations. PotatoRTD contained 37,547 genes and 165,512 transcripts, representing a 3.7-fold increase in transcript isoforms compared with the Potato DM v6.1 annotation (44,851 transcripts) (Table 4). The integration of long and short read data substantially increased the number of multi-isoform genes, with 21,472 loci (57%) encoding more than one isoform, compared to 7,161 in the potato DM v6.1 annotation. On average, each gene had 4.41 transcripts, far higher than potato DM v6.1 annotation (1.36). The potatoRTD also captured extensive exon–intron structures, with over 1.19 million exons and 1.02 million introns, while maintaining consistent transcript quality metrics (N50 = 2,523 bp, average exonic length = 2,234 bp). The characterized genomic regions expanded from 49.8 M bases in Potato DM v6.1 annotation to 82.7 M, which represents a 66% increase while the number of genes has a modest increase of 14% from 32.9 K to 37.5 K. Combined with the statistics showing longer transcripts (55% increase in N90), more exons (3.6 fold increase), our potatoRTD provides a comprehensive view of the transcriptome with higher resolution on all components of transcript structures.

**Table 4:** Statistics of PotatoRTD.

Category	DM v6.1	Iso-seq	RNA-seq	potatoRTD
Genome covered bases	49,825,356	69,303,769	70,932,816	82,686,374
Gene number	32,917	23,448	33,175	37,547
Multi-isoform gene number	7,161	18,552	13,370	21,472
Mono-exon gene number	5,530	2,783	6,105	6,511
Multi-exon gene number	27,387	20,665	27,070	31,036
Transcript number	44,851	145,742	68,038	165,512
Mono-exon transcript number	5,889	15,495	6,996	19,014
Multi-exon transcript number	38,962	130,247	61,042	146,498
Transcript number per gene	1.363	6.216	2.051	4.408
Exon number	258,508	1,095,034	431,997	1,190,244
Exon number per transcript	5.764	7.514	6.349	7.191
Exon average length	277.7226	312.2851	337.5945	310.6675
Intron number	213,657	949,292	363,959	1,024,516
Intron number per transcript	4.764	6.514	5.349	6.19
Intron average length	612.8709	672.168	675.2925	676.1394
Transcript N50	1,989	2,554	2,571	2,523
Transcript N90	944	1,563	1,280	1,468
Transcript average length (exonic)	1,600.71	2,346.36	2,143.51	2,234.10
Transcript total length (exonic)	71,793,526	341,962,831	145,839,797	369,770,082

Similarly, TomatoRTD showed a comparable expansion. The integrated dataset contained 35,942 genes and 156,611 transcripts, with 19,212 multi-isoform genes (53% of loci) and an average of 4.36 transcripts per gene (Table 5). Exon and intron structures were also substantially expanded relative to the tomato M82 annotation, with 1.1 million exons and 0.94 million introns recorded in the final tomatoRTD. Transcript length distribution improved markedly, with N50 reaching 3,002 bp compared to 1,780 bp in the tomato M82 annotation. Comparing the TomatoRTD and the tomato M82 annotation, we observed 91% increase in genome coverage, 123% increase in transcript N90 length and 5.8 fold increase of unique exon numbers.

**Table 5:** Statistics of TomatoRTD.

Category	M82	Iso-seq	RNA-seq	TomatoRTD
Genome covered bases	44,283,595	73,674,580	69,688,679	84,850,936
Gene number	34,619	22,513	33,816	35,942
Multi-isoform gene number	0	17,584	12,656	19,212
Mono-exon gene number	8,449	2,968	7,851	7,945
Multi-exon gene number	26,170	19,545	25,965	27,997
Transcript number	34,619	138,131	66,593	156,611
Mono-exon transcript number	8,449	18,242	9,022	23,441
Multi-exon transcript number	26,170	119,889	57,571	133,170
Transcript number per gene	1	6.136	1.969	4.357
Exon number	160,607	1,033,736	400,883	1,101,280
Exon number per transcript	4.639	7.484	6.02	7.032
Exon average length	275.7264	352.5678	356.3287	349.7878
Intron number	125,988	895,605	334,290	944,669
Intron number per transcript	3.639	6.484	5.02	6.032
Intron average length	622.3709	632.1325	633.1449	635.6607
Transcript N50	1,780	3,053	2,725	3,002
Transcript N90	684	1,628	1,311	1,523
Transcript average length (exonic)	1,279.17	2,638.53	2,145.06	2,459.69
Transcript total length (exonic)	44,283,596	364,462,031	142,846,108	385,214,328

Taken together, both RTDs represent more comprehensive, isoform-resolved reference. The significant increase in multi-isoform loci and transcript diversity provides a robust foundation for downstream analyses, particularly in isoform-level expression quantification and alternative splicing studies.

### Checking the improved AS diversity in new RTDs

**Fig. 3:**
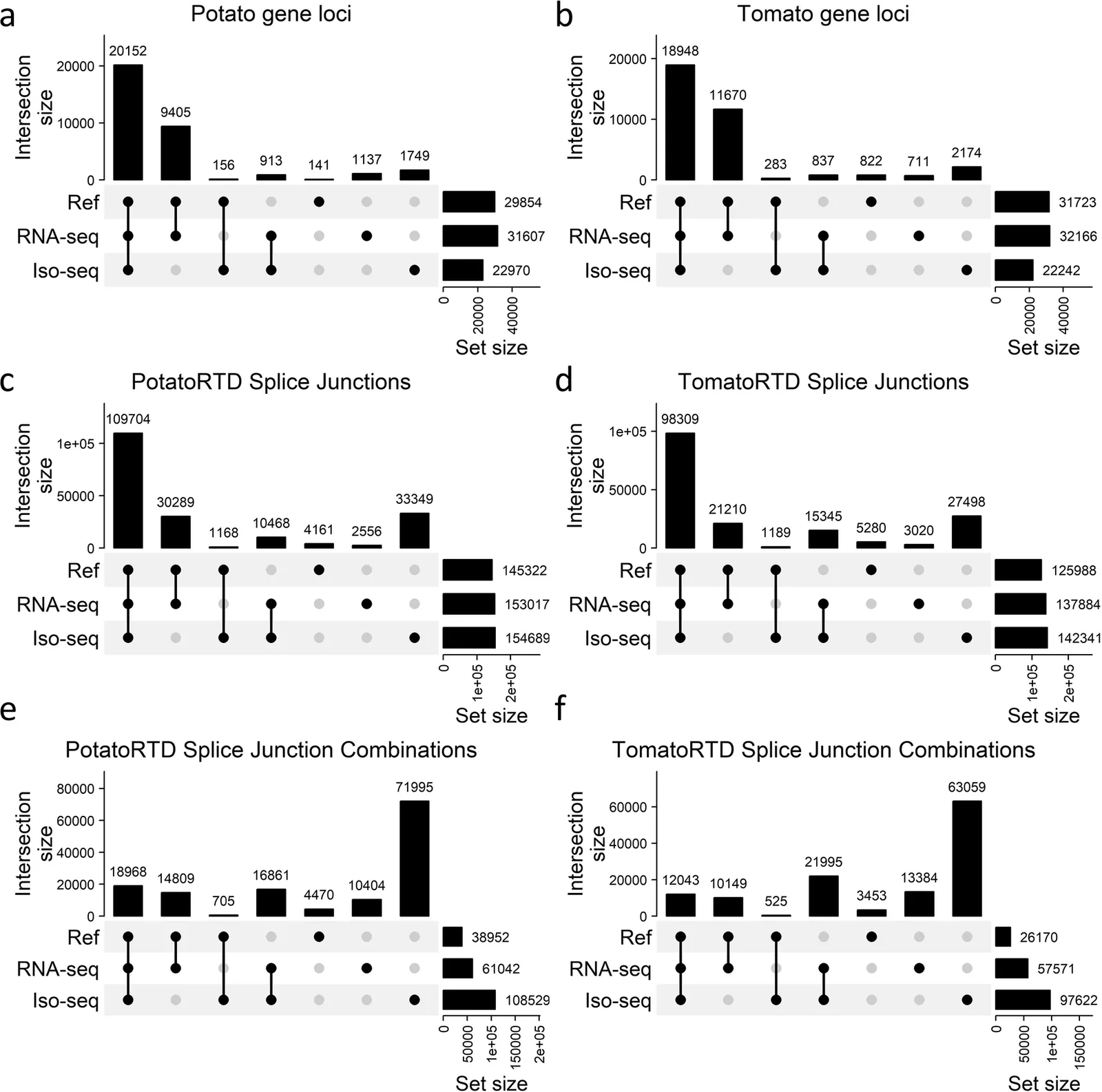
Comparisons of Gene loci and Splice junction diversity between existing annotation (Ref), RNA-seq(short read), and Iso-seq(long read) in PotatoRTD and TomatoRTD.

To assess the contribution of different data sources to transcriptome diversity, we compared splice junctions (SJs) and splice junction combinations (SJCs) detected in PotatoRTD and TomatoRTD (Fig. 3). For PotatoRTD, the numbers of splice junctions detected in long read, short read and potato DM v6.1 annotation are similar between 145 K to 155 K, yielding a total of 191.7 K non-redundant SJs (Fig. 3c). 109.7 K (57.2%) SJs were detected by all three sources of annotation. In terms of unique SJs to each annotation, potato DM v6.1 contributed only 4 K (2.2%), the Iso-seq and RNA-seq assemblies add 46.4 K (24.2%) to the existing transcript annotations.

The differences and diversity at the transcript level are more profound with the number of SJCs starting from 38.9 K in potato DM v6.1 annotation, increased to 61 K from short read assemblies to 108.5 K in Iso-seq assemblies (Fig. 3e). SJCs represent transcript groups where only the differences of the splice junction location and combinations are taken into consideration while the variations at TSS and TES are ignored. Only 4.5 K (3.2%) SJCs out of a total 138.2 K non-redundant SJCs are uniquely represented in the potato DM v6.1 whereas new long- and short-read assemblies add 99.3 K (71.8%) SJCs to the final PotatoRTD. The Iso-seq assembly yielded a striking number of unique SJCs, with 72 K (52%) absent from other annotations. This demonstrates that long read sequencing uncovers greater transcript diversity that previous technologies could not detect.

The increase of AS events and transcript diversity is even more pronounced in TomatoRTD. Iso-seq and short read assemblies add 45.9 K (26.7%) novel SJs to the existing tomato M82 annotation, yielding a total of 171.9 K non-redundant SJs (Fig. 3d). At the transcript level, long- and short-read assemblies adds 98.4 K (79%) novel SJCs to the total of non-redundant 124.6 K SJCs (Fig. 3f).

We also observed a higher percentage of overlaps between the short-read assemblies and existing annotations. This could be partially due to the current annotations have used RNA-seq sequencing for improvement or updates. The unique splice junctions and transcripts identified by Iso-Seq sequencing highlight the distinct advantages of long-read technologies, which enable the direct observation of alternative splicing and transcript diversity that were previously inaccessible using sequencing approaches of previous generations.

However, we also observed that long-read sequencing captures around a quarter less gene loci compared to existing annotations (23% less in Potato and 30% less in Tomato) (Fig. 3a, b), which is consistent with our previous observations that the long-read sequencing has significant limitations on the gene coverage^16,18^. Thus, despite the advantage long-read sequencing offers in terms of transcript integrity and accuracy, short read sequencing still has great values in the coverage for gene discoveries. The sequencing effort also helps to uncover a few thousand transcribed genomic regions that are missing in the existing annotation. In the PotatoRTD, RNA-seq and Iso-seq data contributed 1,137 and 1,749 novel gene loci, respectively. Similarly, in the tomatoRTD, RNA-seq identified 711 novel gene loci, whereas Iso-seq contributed 2,174 novel gene loci. It is also worth noting that, because it is not feasible to sample every possible condition at sufficient depth to detect every transcript, low-expression or highly condition-specific genes may still be incompletely represented in the RTDs. Continued efforts will therefore be needed to further improve and expand these resources.

### Checking the definition of transcript boundaries in the new RTDs

**Fig. 4:**
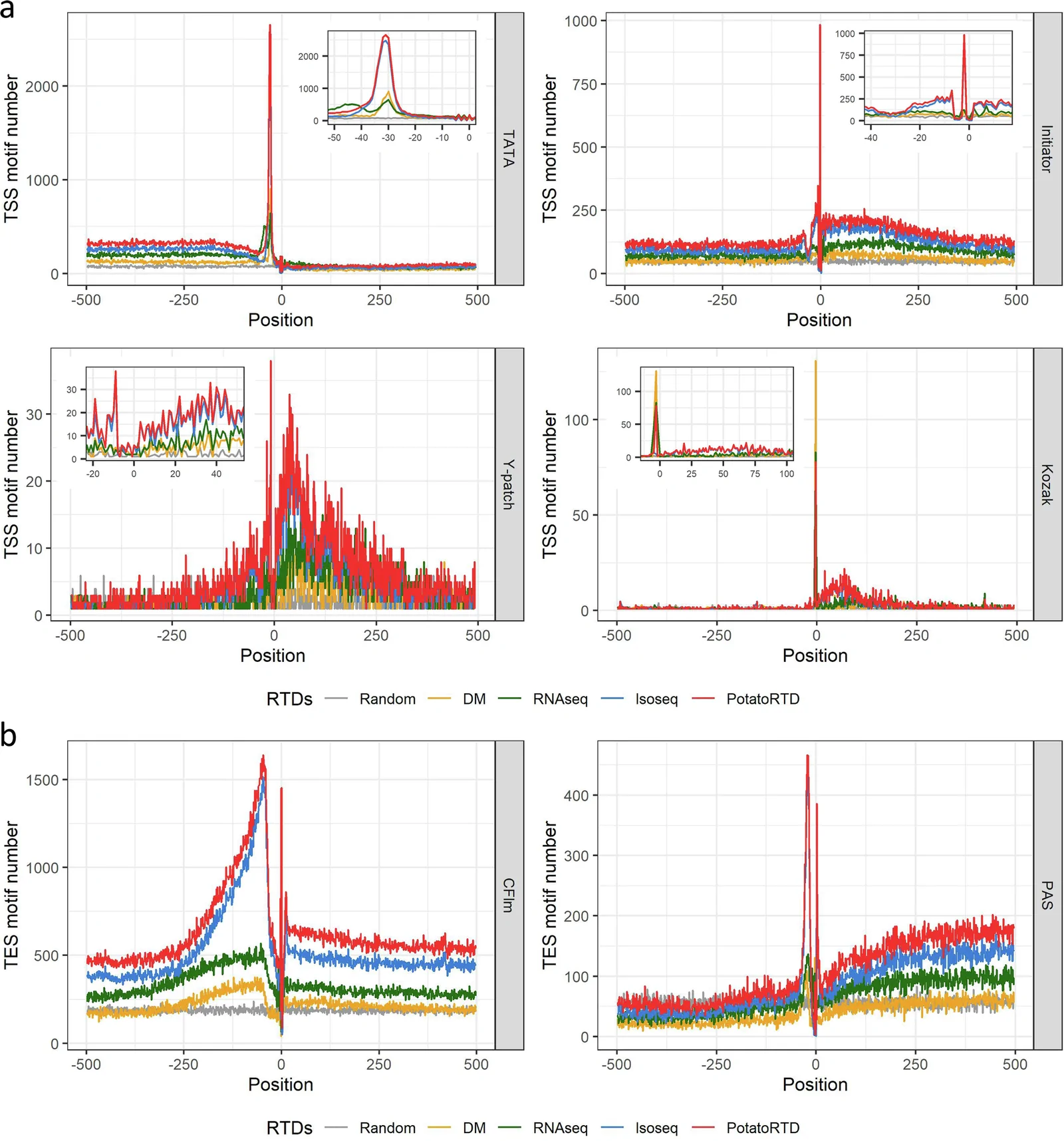
Motif enrichment around TSS and TES in potato annotations. Distribution of transcription start site (TSS) motifs (TATA, Initiator, Y-patch, Kozak) and transcription end site (TES) motifs (CFIm, PAS) was analysed in PotatoRTD (red) to potato DM v6.1, Iso-seq RTDs, RNA-seq RTDs, and random controls. PotatoRTD showed the strongest motif number precisely at annotated TSS and TES, indicating improved accuracy of transcript boundary definition compared to other annotations.

**Fig. 5:**
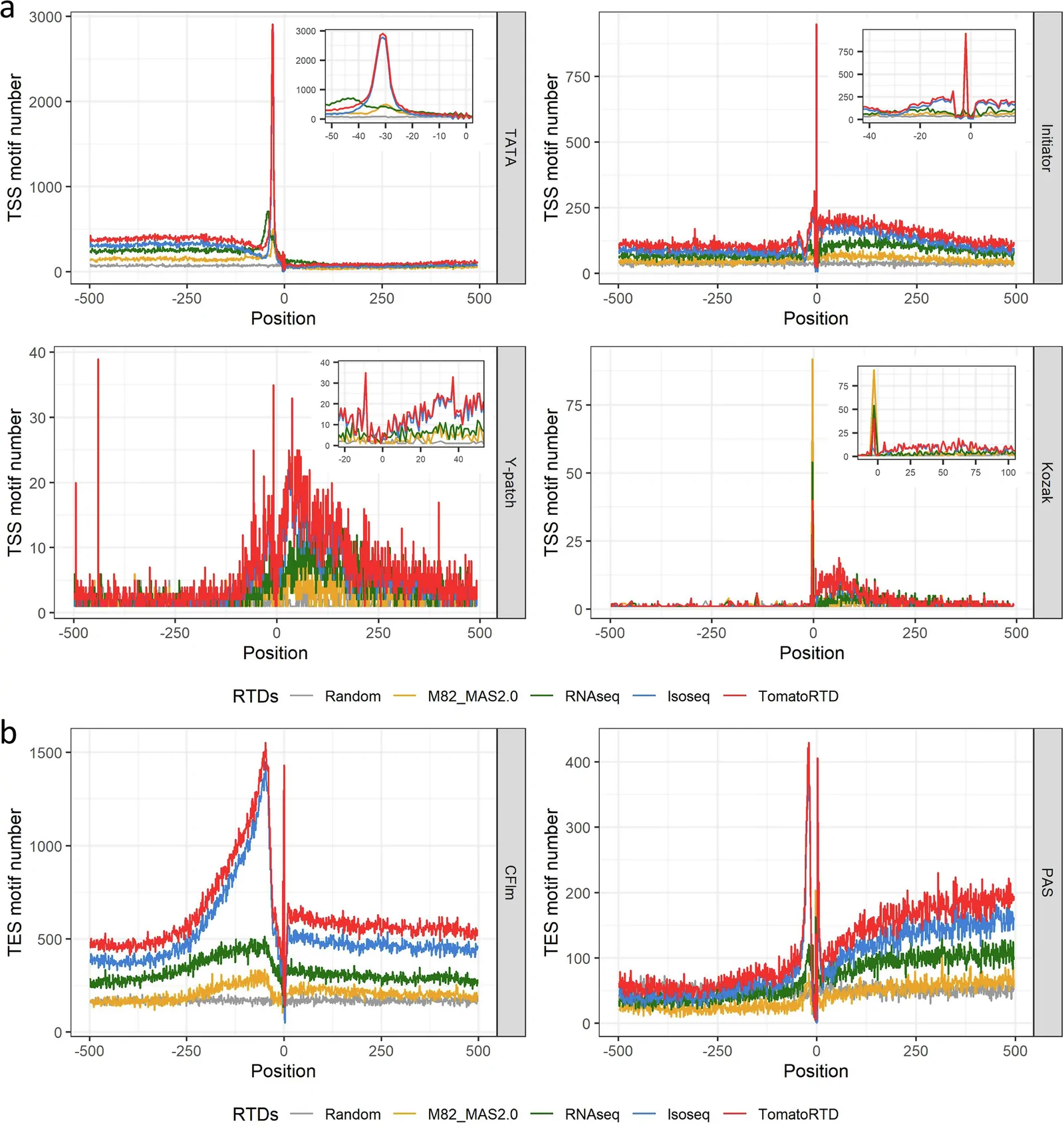
Tomato TSS and TES motif. Distribution of transcription start site (TSS) motifs (TATA, Initiator, Y-patch, Kozak) and transcription end site (TES) motifs (CFIm, PAS) was analysed in TomatoRTD (red) to tomato M82 annotation, Iso-seq RTDs, RNA-seq RTDs, and random controls. TomatoRTD showed the strongest motif number precisely at annotated TSS and TES, indicating improved accuracy of transcript boundary definition compared to other annotations.

An accurate TSS and TES is key for modification of gene expression using CRISPR or other methods. To assess the accuracy of TSSs/TESs, we examined the total number of known sequence motifs around TSS and TES across different transcript annotations. For TSS, we analysed canonical promoter-associated motifs including TATA box^31^, Initiator^32^, Y-patch^33^, and Kozak^32^. For TES, we evaluated enrichment of polyadenylation-associated motifs CFIm^34^ and PAS^35^. The PotatoRTD and long read Iso-seq assembly show the highest peak at 0 position for all motifs related to TSS and TES, except the Kozak motif, indicating the PotatoRTD and the long-read Iso-seq assembly likely detects more biologically genuine TSS/TES sites. We observed the same improvement using our pipeline in the ***Arabidopsis*** study^16^. Kozak sequence is a motif related to protein translation initiation site. A high peak around zero indicates a significant number of transcripts missing 5′ UTR regions. Figure 4a shows that the DM v6.1 annotation has the highest peak around zero, followed by short read RNA-seq assemblies with long read lso-seq assembly has the lowest peak among all annotations around zero and much higher values from 0-250nt, which indicates the Iso-seq assembly includes more transcripts with increased length of 5′ UTR regions. As a result, the PotatoRTD shows a reduced peak around zero and increased number of transcripts with 5′ UTRs. A similar pattern was observed in tomato (Fig. 5), where TomatoRTD consistently outperformed the tomato M82 reference RTD, Iso-seq RTD, and RNA-seq RTD. TomatoRTD showed narrow and highly enriched motif peaks at both TSS and TES, reflecting improved precision in transcript boundary annotation.

### Data use-case scenarios

We present two use cases here, using PotatoRTD as an example, to demonstrate the value and analytical improvements provided by the proposed dataset for transcriptome analysis.

In the first example, we followed a previously published study^36^ that investigated the regulation of tuberization initiation and axillary bud activation. RNA sequencing was performed on hooked and swelling stolons of wild-type and the transgenic *StGERMIN3* OE line 66. Transcript abundances in PotatoRTD were quantified from RNA-seq reads using Salmon. The analysis reduced gene model ambiguity, particularly among the closely related GLP genes and provided more accurate splice-junction counts across stolon stages. With this analysis, a large scale developmental reprogramming was detected when stolons changed from hooked to swelling stage, with 5,409 differentially expressed transcripts in wild type (2,011 up and 3,398 down) and 3,722 in GERMIN3 overexpression line (1,451 up and 2,271 down), of which 3,233 transcripts were shared. At the same time, PotatoRTD helped to identify a small set of genotype-specific changes: 10 DE transcripts in the hooked stage (all higher in OE) and 50 in the swelling stage (46 higher and 4 lower). These included GERMIN3 itself, a second GLP, several cell wall–related kinases, and an ARF GTPase. The isoform-level information in PotatoRTD was important to separate paralogous GLPs and to distinguish isoforms at different stages, which improved the statistical power and made the biological interpretation more reliable. Altogether, PotatoRTD allows accurate isoform-aware DE analysis, and it supported the conclusion that GERMIN3 promotes meristem activation and tuber initiation but does not change the whole tuberisation program.

In the second use case, we analysed a dataset that aims to understand the transcriptomic reprogramming under biotic stress in potato^37^. In this experiment, spore suspension of *P. infestans* isolate W9928C was used to infect leaves of doubled monoploid potato (*Solanum tuberosum* group Phureja DM 1-3 516 R44) (hereafter referred to as DM). Four independent biological replicates of leaves were sampled from infected DM microshoots at 0 h (T0) and 48 h (T48). The RNA-seq reads were trimmed using fastp and mapped to reference transcriptomes: Potato DM v6.1 and PotatoRTD for quantification using Salmon 1.9.0 in mapping-based mode. The outputs of transcript quantification from Salmon were analysed using 3D RNA-seq App^38^ in default settings.

**Fig. 6:**
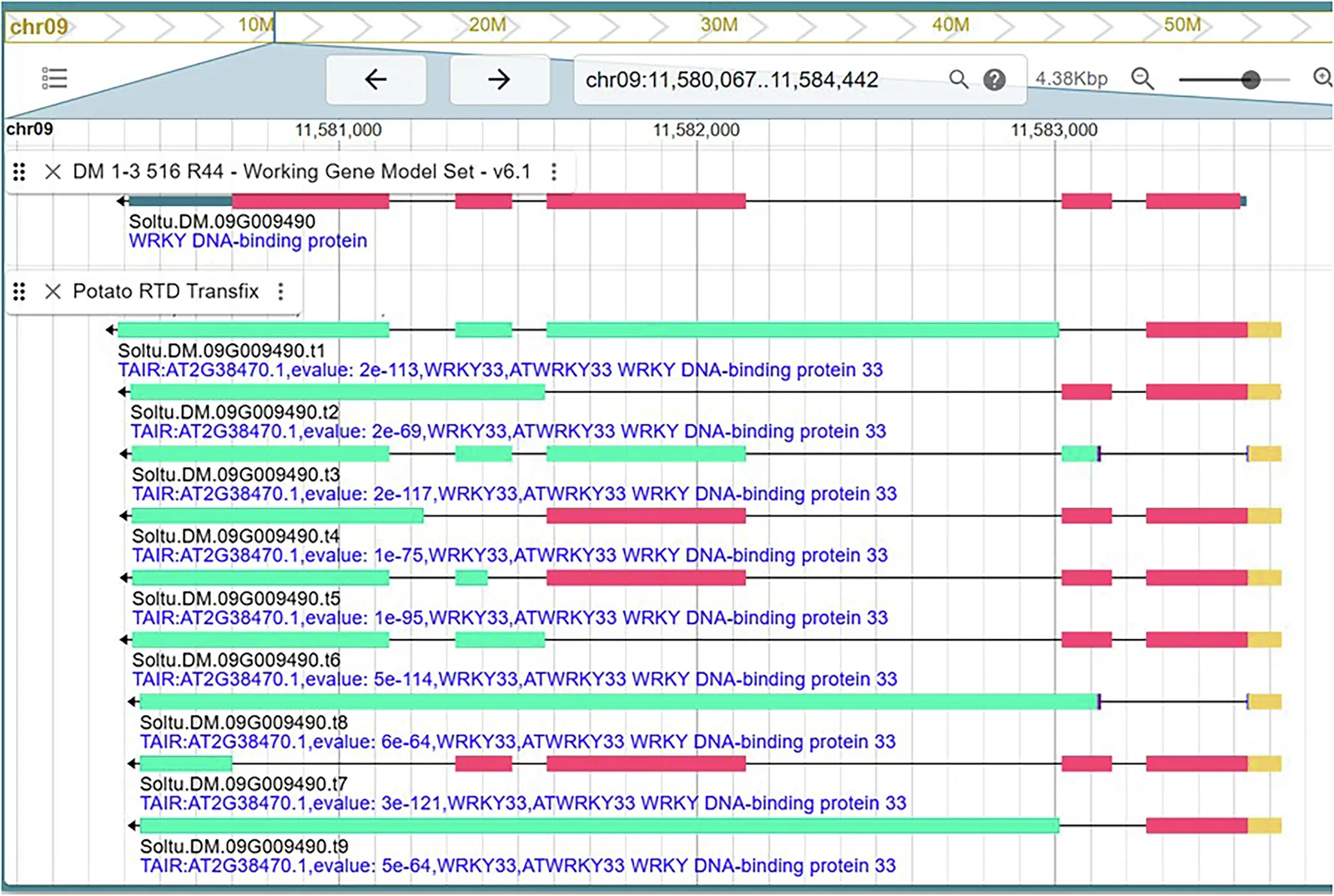
Nine transcript isoforms captured in PotatoRTD for a well characterized alternative spliced gene WRKY33.

**Fig. 7:**
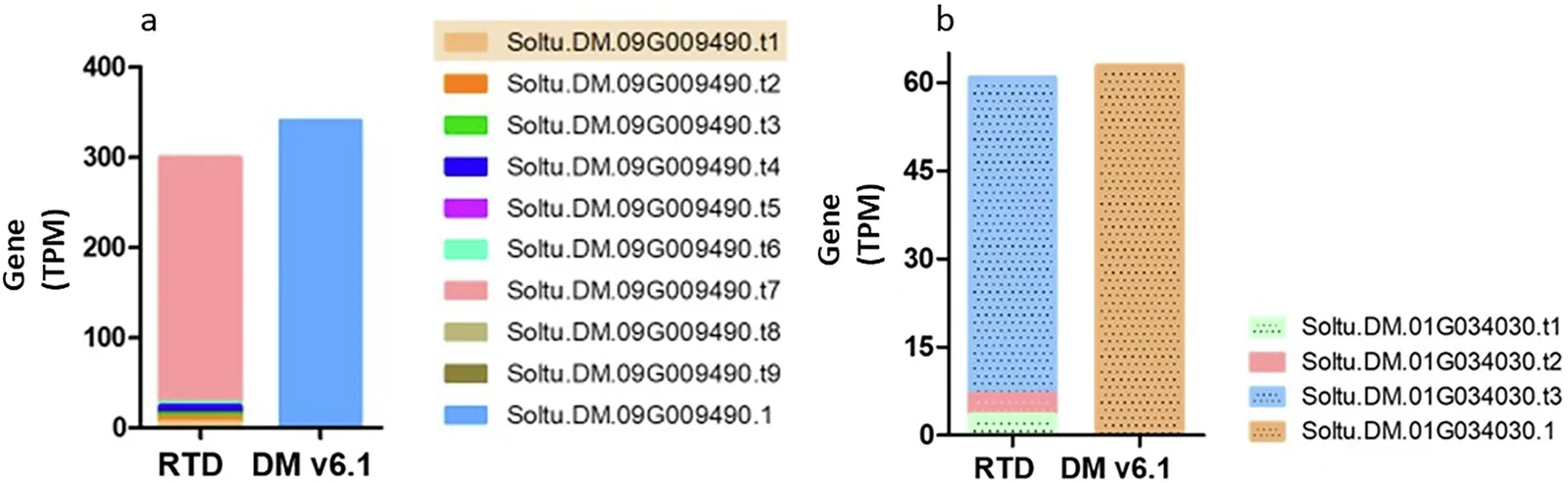
Transcript isoform quantification is enabled when using PotatoRTD as reference when multiple isoforms are characterized in PotatoRTD while there is only a single isoform in DM v6.1 annotation.

The average mapping rate to PotatoRTD (94.05%) is 7.6% higher compared to potato DM v6.1 (86.47%), which is consistent with the improved mapping rates observed in our previous studies due to the completeness of our transcriptome annotations^15,17–19^. We also identified substantially more differentially expressed transcripts (DETs) when using PotatoRTD (29,966) compared to potato DM v6.1 (17,195). PotatoRTD also allows the investigation of target genes with a greater resolution. For instance, a total of 9 transcripts were identified for Soltu.DM.09G009490 encoding a WRKY DNA-binding protein using Potato RTD whereas the DM v6.1 transcriptome indicated only one transcript (Fig. 6 and Fig. 7a). *Soltu.DM.09G009490* is homologous to ***Arabidopsis*** **WRKY33**, a conserved defence-related transcription factor whose regulation by alternative splicing is well established. The expanded isoform repertoire revealed here indicates that WRKY33-like splicing complexity is conserved in potato but was previously under-annotated.

Similarly, for Soltu.DM.01G034030 encoding for MAPK, 3 transcripts were identified with potato RTD and only one with the DM v6.1 transcriptome (Fig. 7b).

In addition to the repository data, our dataset is also available for users to download and visualise at https://ics.hutton.ac.uk/potatortd/index.html for potato and https://ics.hutton.ac.uk/tomatortd/index.html for tomato.

## Data Availability

All sequencing data (PacBio Iso-seq and Illumina) have been deposited at the Sequence Read Archive (SRA) under the Bioproject number: PRJNA1412869 (potato samples: https://www.ncbi.nlm.nih.gov/bioproject/PRJNA1412869) and PRJNA1406150 (tomato samples: https://www.ncbi.nlm.nih.gov/bioproject/PRJNA1406150).The transcript sequences, transcript annotation in GTF format, protein translation in fasta format, functional annotations from InterproScan and Panzer are available in Zenodo under 10.5281/zenodo.20160822 (https://zenodo.org/records/20160822).

## Code availability

The code for analysing the RNA-sequencing and PacBio Iso-seq data as well as the code for construction of the genome browsers are available on GitHub: https://github.com/wyguo/potatoRTD_tomatoRTD.
